# Conditional Knockouts of Interphotoreceptor Retinoid Binding Protein Suggest Two Independent Mechanisms for Retinal Degeneration and Myopia

**DOI:** 10.1167/iovs.65.6.32

**Published:** 2024-06-21

**Authors:** Tatiana E. Getz, Micah A. Chrenek, Jack T. Papania, Debresha A. Shelton, Shanu Markand, P. Michael Iuvone, Zbynek Kozmik, Jeffrey H. Boatright, John M. Nickerson

**Affiliations:** 1 Emory University, Department of Ophthalmology, Atlanta, Georgia, United States; 2 Kirksville College of Osteopathic Medicine, A.T. Still University, Kirksville, Missouri, United States; 3 Institute of Molecular Genetics of the ASCR, Prague, Czech Republic; 4 Atlanta Veterans Administration Center for Visual and Neurocognitive Rehabilitation, Decatur, Georgia, United States

**Keywords:** retinoids, retinoid binding protein, protein

## Abstract

**Purpose:**

Interphotoreceptor retinoid-binding protein's (IRBP) role in eye growth and its involvement in cell homeostasis remain poorly understood. One hypothesis proposes early conditional deletion of the IRBP gene could lead to a myopic response with retinal degeneration, whereas late conditional deletion (after eye size is determined) could cause retinal degeneration without myopia. Here, we sought to understand if prior myopia was required for subsequent retinal degeneration in the absence of IRBP. This study investigates if any cell type or developmental stage is more important in myopia or retinal degeneration.

**Methods:**

IBRP^fl/fl^ mice were bred with 5 Cre-driver lines: HRGP-Cre, Chx10-Cre, Rho-iCre75, HRGP-Cre Rho-iCre75, and Rx-Cre. Mice were analyzed for IRBP gene expression through digital droplet PCR (ddPCR). Young adult (P30) mice were tested for retinal degeneration and morphology using spectral-domain optical coherence tomography (SD-OCT) and hematoxylin and eosin (H&E) staining. Function was analyzed using electroretinograms (ERGs). Eye sizes and axial lengths were compared through external eye measurements and whole eye biometry.

**Results:**

Across all outcome measures, when bred to IRBP^fl/fl^, HRGP-Cre and Chx10-Cre lines showed no differences from IRBP^fl/fl^ alone. With the Rho-iCre75 line, small but significant reductions were seen in retinal thickness with SD-OCT imaging and postmortem H&E staining without increased axial length. Both the HRGP-Cre+Rho-iCre75 and the Rx-Cre lines showed significant decreases in retinal thickness and outer nuclear layer cell counts. Using external eye measurements and SD-OCT imaging, both lines showed an increase in eye size. Finally, function in both lines was roughly halved across scotopic, photopic, and flicker ERGs.

**Conclusions:**

Our studies support hypotheses that for both eye size determination and retinal homeostasis, there are two critical timing windows when IRBP must be expressed in rods or cones to prevent myopia (P7-P12) and degeneration (P21 and later). The rod-specific IRBP knockout (Rho-iCre75) showed significant retinal functional losses without myopia, indicating that the two phenotypes are independent. IRBP is needed for early development of photoreceptors and eye size, whereas Rho-iCre75 IRBP^fl/fl^ knockout results in retinal degeneration without myopia.

Interphotoreceptor retinoid-binding protein (IRBP) is a highly conserved and abundant protein that plays a critical role in the transport of retinoids between the outer segments of photoreceptor cells and retinal pigment epithelium (RPE).^1^ It is a glycoprotein that is primarily synthesized and secreted by photoreceptor cells^2^ and secreted into the interphotoreceptor matrix (IPM) into the subretinal space.^3^^–^^5^ IRBP binds to retinoids, including all-trans-retinol and 11-cis-retinal, among other retinoids and fatty acids, buffering them during the visual process.^6^^–^^10^ IRBP mutations cause RP66, which is severe retinal degeneration with myopia.^11^^,^^12^ Alterations in IRBP gene expression or function have been associated with inherited retinal diseases,^11^^–^^14^ uveitis,^11^^,^^12^^,^^15^^,^^16^ and eye size.^17^ Understanding IRBP's posited multiple functions may provide insights into the pathogenesis and treatment of all these diseases.

IRBP is expressed precociously early (E12),^18^^–^^21^ well before the visual cycle starts to function in development, leading to the conjecture that IRBP may a play role in ocular development, but distinct from its role in the visual cycle. The eyes of mice with a traditional germline knockout of IRBP (IRBP^ko/ko^) develop extreme myopia by postnatal day 8, with a myopic shift of 15 diopters compared to wildtype, and having eyes of significantly longer axial length (AL).^22^^,^^23^ A late-onset, slowly progressing retinal degeneration starts abruptly at postnatal day 25,^22^^,^^24^^–^^28^ and may or may not be associated with the early developmental deficiencies. It thus has been hypothesized that IRBP plays two distinct roles, one in early development of eye size and a second role later in the visual cycle and in maintaining retinal homeostasis, integrity, and function.

Here, we sought to understand if prior myopia was required for subsequent retinal degeneration in the absence of IRBP. In addition, we sought to understand if the expression of IRBP was required at specific times during development and maturation of the retina, and if IRBP expression was required in any specific cell types. In particular, this study aims to determine whether IRBP expression in one cell type is more critical for the prevention of myopia and expression in a different cell type at a different time is required to prevent retinal degeneration. Using the Cre-lox system, IRBP can be selectively removed from specific cell types at specific time points. For these experiments, we created, to our knowledge, the first IRBP^fl/fl^ mouse (generation of this line is detailed below). We used a strategy of conditional knocking out of the IRBP gene by using a set of Cre-driver lines with Cre expression controlled by promoters that are cell-type and timing-specific in expression. This experiment used HRGP-Cre, Chx10-Cre, Rho-iCre75, HRGP-Cre+Rho-iCre75, and Rx-Cre as the Cre driver systems. HRGP-Cre targets cone photoreceptors^29^ (which are born and express IRBP early in development^30^), whereas Rho-iCre75 is selectively expressed in rod photoreceptors^31^ (which are born late in development and eventually express IRBP heavily). Rho-iCre75 expresses Cre later in eye development reaching functional levels at about P11,^31^^,^^32^ because rod photoreceptors do not develop until late in eye growth and the rhodopsin promoter is not activated until then. Therefore Rho-iCre75 will cause a late deletion in comparison to the various Cre-driver strains. The HRGP-Cre+Rho-iCre75 was used to test rod and cone photoreceptors in combination, as this line knocks out IRBP in both rods and cones. Chx10-Cre targets most retinal progenitor cells starting at E10,^33^ but bipolar cells are the main cell type in which Cre is expressed. Rx-Cre targets the entire neural retina early in development with substantial Cre in all retinal cells.^34^^,^^35^ Outcomes from each of the five aforementioned Cre driver lines, bred to the IRBP^fl/fl^ mouse that we created, were evaluated at approximately postnatal day 30, a time point when both myopia and retinal degeneration were robustly evident in the germline IRBP^ko/ko^ mouse. Each line was tested for IRBP gene expression, retinal function (electroretinogram [ERG]), in vivo and post-mortem morphology (spectral-domain optical coherence tomography [SD-OCT] and hematoxylin and eosin [H&E]), and eye size. Our findings showed that early severe myopic changes were not a prerequisite for later retinal degeneration, suggesting two distinct and independent functions of IRBP.

## Methods

### Animal Studies

This research was approved by the Emory Institutional Animal Care and Use Committee (IACUC) and adheres to ARVO Statement for the Use of Animals in Ophthalmic and Vision Research. All mouse outcomes were measured and collected at approximately postnatal day 30. The genetic background of these mice was C57Bl/6J. All mice were fed ad libitum standard mouse chow diet (Lab Diet 5053; PMI Nutrition Inc., LLC, Brentwood, MO, USA). Adult mice were euthanized by CO_2_ for 5 minutes, followed by cervical dislocation.

### Breeding Scheme

To generate a floxed (see Supplementary text file) allele of IRBP, we attempted to do this using traditional methods using a vendor (Genoway, Lyon, France). The resulting mouse had a single LoxP site inserted on the 3′ end of the first exon, as well as 2 short spacer sequences, and a single FRT site. We inserted the second LoxP site on the 5′ side of the first exon using CRISPR technology at the Emory Transgenic Mouse Facility. A map of the locations of the LoxP sites at -643 bp upstream from the transcription start site and 3117 bp downstream from the transcription start site in the first intron are indicated in a sequence map in the Supplementary Figure S1. Supplementary Figure S2 shows the amino acid structure of the repeated elements in IRBP and the locations of the deleted region of the gene (approximately 3500 nucleotides in length). Once the sequence between the loxP sites is deleted, residual parts of the IRBP gene lack a functional promoter, lack cis-elements, and lack a functional transcription start site. The resulting mice were bred to homozygosity, the region sequenced, and confirmed to have normal function. The sequence of the resulting IRBP floxed allele is provided as supplementary data in a text file in the Supplementary files. We will make the IRBP^fl/fl^ mouse available from Jackson Labs, as soon as they accept it. HRGP-Cre mice (Jax stock #032911) were provided by Yun Z. Le at the University of Oklahoma and targets the cone photoreceptors.^29^ HRGP-Cre mice bred with the IRBP^fl/fl^ mice to create 50% experimental HRGP-CRE IRBP^fl/fl^ and 50% control IRBP^fl/fl^ mice. Chx10-Cre (Jax stock #005105)^33^ targets most retinal progenitor cells and were bred with IRBP^fl/fl^ mice to have litters 50% Chx10-Cre IRBP^fl/fl^ mice and 50% IRBP^fl/fl^ mice. Rho-iCre75 mice (Jax stock #015850)^31^ targets rod photoreceptors and were bred with IRBP^fl/fl^ mice to create litters 50% Rho-iCre75 IRBP^fl/fl^ and 50% IRBP^fl/fl^ mice. Rx-Cre targets early progenitor cells and the entire neural retina.^34^ Rx-Cre mice were bred with IRBP^fl/fl^ mice to give litters 50% Rx-Cre IRBP^fl/fl^ and 50% IRBP^fl/fl^. The double knockout mice were bred with HRPG-Cre IRBP^fl/fl^ and Rho-iCre75 IRBP^fl/fl^ mice to create a HRGP-Cre+Rho-iCre75 IRBP^fl/fl^ mouse strain, (which lacks the IRBP gene in almost all rod and cone photoreceptors but contains the IRBP gene in all other cells).

### Electroretinograms

Mice were dark adapted overnight the day before ERGs were conducted, as previously described.^36^^,^^37^ Under infrared lighting, mice were given intraperitoneal (IP) injections of 100 mg/kg ketamine and 15 mg/kg xylazine (ketamine; KetaVed from Vedco Inc., St. Joseph, MO, USA; AnaSed from Akorn, Lake Forest, IL, USA). After the mice were anesthetized, proparacaine eye drops (1%, Akorn Inc.) were applied for 2 minutes. Tropicamide eye drops (1%, Akorn Inc.) were applied for 5 minutes to ensure dilated pupils. The mice were then placed on the Diagnosys Celeris System (Diagnosys, LLC, Lowell, MA, USA). The electrodes were placed in contact with the eyes and a full field scotopic ERG was performed. After scotopic ERGs, the mice were light adapted for 10 minutes before photopic and flicker ERGs were collected.^38^ Afterward, the mice were placed on a heating pad (37°C) for recovery from anesthesia. Both eyes were imaged and analyzed and then averaged together for each mouse.

### SD-OCT Imaging

Mice were anesthetized, and eye drops were applied, as previously described.^22^^,^^23^ A Micron IV SD-OCT system with a fundus camera (Phoenix Research Labs, Bend, OR, USA) was used to capture in vivo fundus and OCT images. The camera was centered around the optic nerve so that the fundus was clearly visible. A circular scan 100 microns from the optic nerve was collected, and averaged across 50 frames. The OCT images were analyzed for total retinal thickness and photoreceptor thickness through Adobe Photoshop CS6 (Adobe Systems Inc., San Jose, CA, USA) with the observer masked to the genotype. Both eyes were imaged and analyzed, and then averaged together to create one data point per mouse.^38^

### Whole Eye Biometry

Mice were anesthetized, and eye drops were applied, as described above. Refresh Tears were then applied on the left eye to keep the eye from drying out. Using a Bioptigen R4310 deep imaging SD-OCT System (Leica Microsystems, Durham, NC, USA), whole eye biometrical images were taken on the left eye only. Images were analyzed for the following parameters: central corneal thickness (CCT), anterior chamber depth (ACD), lens thickness (LT), vitreous chamber depth (VCD), retinal thickness (RT), and total axial length (TAL).^23^

### Gene Expression

Mice were euthanized through 5 minutes of CO_2_ exposure followed by cervical dislocation. The right eye was enucleated, and a dissection was performed to isolate the retina. The retina was immediately frozen in dry ice and RNA isolation was performed with the Qiagen RNeasy RNA extraction kit. The RNA was then used to create cDNA with the Qiagen QuantiTect cDNA synthesis kit.

IRBP gene expression was quantified by digital droplet PCR (ddPCR) using an AutoDG droplet generator, QX200 droplet reader, and associated supplies from Biorad (Hercules, CA, USA). Biorad ddPCR Supermix for Probes (no dUTP) was used (cat# 1863024) with gene expression probe-based assays (assays presented in the Table). Expression of target genes were measured relative to HPRT in the same reactions. PCR conditions were as follows: 1 cycle of 95°C for 10 minutes; 40 cycles of 95°C for 30 seconds, 60°C for 60 seconds; 1 cycle of 98°C for 10 minutes; and then on hold at 4°C.

**Table.** Assays Used for ddPCR

Gene	Fluor	Vendor	AssayID
IRBP	FAM	IDT	Mm.PT.58.8868329
HPRT	HEX	IDT	Mm.PT.39a322214828

### Eye Metrology

After euthanasia, the eyes were removed using forceps; and any remaining ocular tissue and muscle were removed. The right eye was then placed on an A-160 analytical balance (Denver Instruments; Fisher Scientific, Pittsburgh, PA, USA) to determine eye weight. External eye dimensions were acquired through a Keyence IM-6145 system (Keyence Inc., Schaumburg, IL, USA). Eyes were measured for equatorial width (W), AL, and roundness (R).^22^

### Sectioning and Histology

The left eye was enucleated and processed for histology using a freeze substitution method.^39^ This was done by placing the eye in 10 mL of dry-ice cooled methanol with 3% acetic acid and exchanging water for methanol in a −80°C freezer for 4 or more days. Afterward, the samples were brought to room temperature for 2 hours, the methanol and any residual water in the eye were exchanged through 4 baths for 20 minutes each: 2 for 100% methanol baths, followed by 2 for 100% xylene baths. The eyes were then incubated in a paraffin bath for 1 hour and then a second paraffin bath overnight at 57°C. After, the eyes were mounted in paraffin cassettes. The eyes were then cut into a series of 5-micron thick sagittal sections, including both the optic nerve head as well as the center of the cornea. Sections were stained with H&E to observe retinal morphology. Outer nuclear layer (ONL) nuclei were manually counted in 100-micron regions every 500 microns from the optic nerve. Retinal arc lengths were obtained through ImageJ by following the inner plexiform layer through the entire length of the retina.^22^ The retinal arc length is the cumulative length measured along the curved path of the inner plexiform layer (IPL). We measured at intervals of every 50 microns, starting from the far peripheral tip of the retina, tracing the perimeter of the IPL through the optic nerve head (ONH), and continuing to the opposite far peripheral point of the retina. These measurements are performed on H&E stained sections cut in a superior-inferior plane, passing through the center of the cornea and ONH.

### Immunostaining

Eyes were enucleated and processed through the freeze substitution method, and then embedded into paraffin cassettes as above. Samples were then cut into 5-micron sections and mounted to slides. Slides were deparaffinized through a series of 5 steps of xylene for 2 minutes. They were transferred into an ethanol rehydration series (100%, 90%, 80%, 70%, 60%, and 50%) and then Tris-Buffered Saline (TBS) for 2 minutes each. A Sequenza staining system 73310017 (Epredia, Kalamazoo, MI, USA) was used to stain the samples. Slides were blocked for 30 minutes with 2.5% normal donkey serum in TBS at room temperature and then a primary antibody was incubated for 60 minutes at room temperature. The primary antibodies used were IBA-1, ab178847 (Abcam, Cambridge, MA, USA) and GFAP, DAKO Z0334 (Agilent, Santa Clara, CA. ISA). Slides were washed with TBS with 0.1% Tween-20 (TBST) twice for 5 minutes, and then incubated in secondary antibody for 60 minutes at room temperature. The secondary antibody used was donkey anti-rabbit 647 A32795 (Life Technologies, Carlsbad, CA, USA). They were then washed twice with TBST for 5 minutes and then stained with Hoescht 33342 (Abcam, Cambridge, MA, USA) for 10 minutes at room temperature. Slides were washed with TBS for 5 minutes and mounted with Vectashield vibrance (Vector Labs Inc., Burlingame, CA, USA) before coverslipping. Imaging was performed with a Nikon Ti2 microscope with A1R confocal imager.

### Statistical Analysis

Sample sizes per group are indicated in figure legends. In most cases, N was greater than 6 and as high as 32. We did not separate male from female subjects in the present figures or calculations, as no trends were noticed for any sex-based differences. In most cases, a roughly equal number of male and female subjects were used. Statistical analysis was performed with Prism, version 9.1 (GraphPad Software, Inc., La Jolla, CA, USA) on a Mac OS version 11.6.8. All data are reported as mean ± standard deviation. All comparisons were made with 1-way or 2-way analysis of variance (ANOVA) and Tukey's multiple comparisons test (sometimes with a Welch correction as appropriate) and indicated in each figure. The ANOVA tests allowed us to determine whether there were any statistically significant differences between the means of the groups being compared. Tukey's post hoc test was then used to identify which specific groups differed from each other while controlling for familywise error rate. The Welch correction was applied when the assumption of homogeneity of variances was violated to provide a valid statistical inference. We did not delete any potential outliers. *P* values < 0.05 were considered to be statistically significant.

## Results

**Figure 1.**
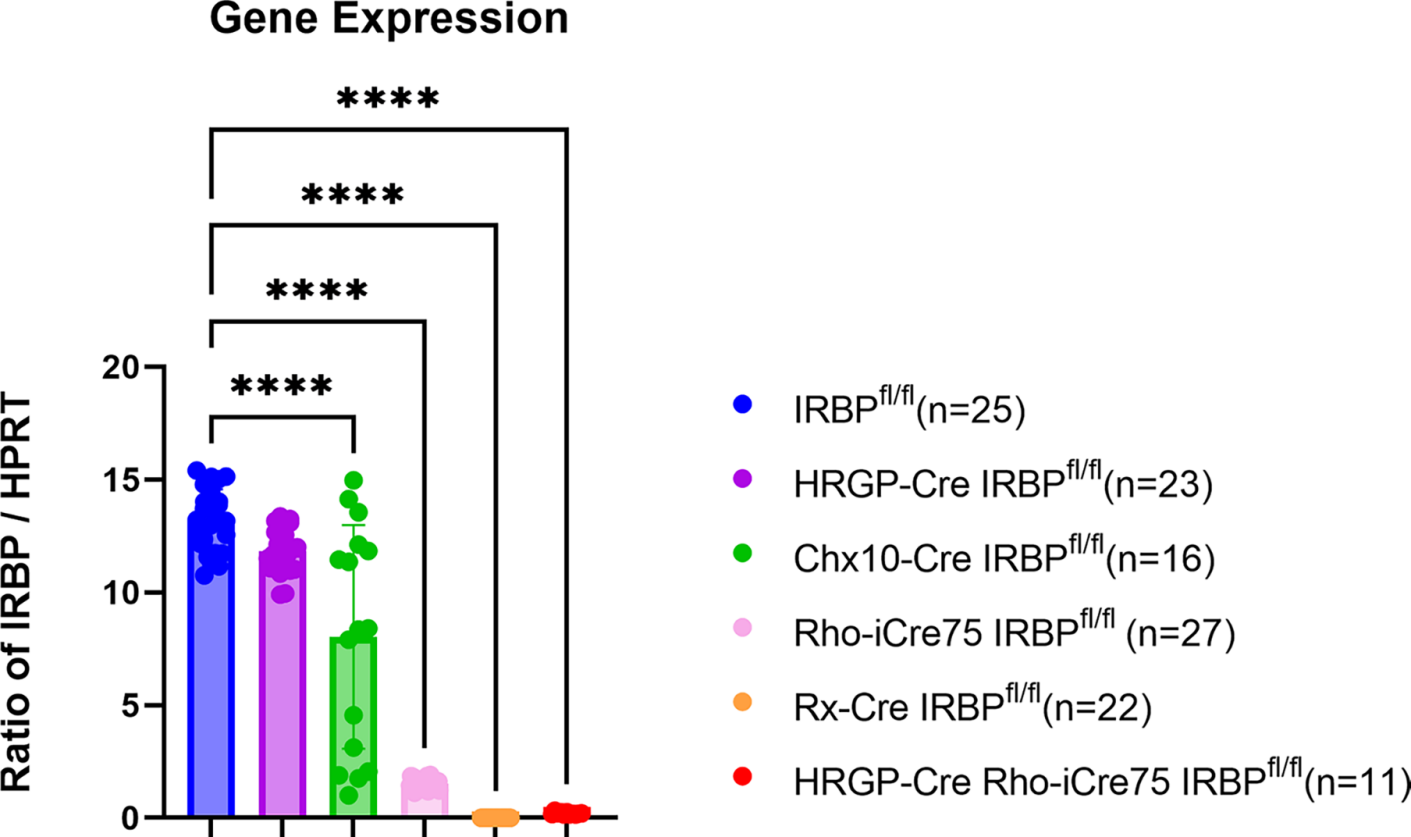
Gene expression confirming loss of IRBP. Isolated retinas were assessed for IRBP gene expression. IRBP^fl/fl^ control mice show normal levels of IRBP expression. HRGP-Cre IRBP^fl/fl^ mice showed no significant difference. Chx10-Cre IRBP mice had a slight decrease. As expected, Rho-iCre75 IRBP^fl/fl^, HRGP-Cre Rho-iCre75 IRBP^fl/fl^, and Rx-Cre IRBP^fl/fl^ have a substantial loss of IRBP expression, validating the use of these Cre constructs in our further experiments. One-way ANOVA with Dunnett's multiple comparison's test. * Represents *P* < 0.05; ** represents *P* < 0.01; and *** represents *P* < 0.001. Sample size = IRBP^fl/fl^ (*n* = 25), HRGP-Cre IRBP^fl/fl^ (*n* = 23), Chx10-Cre IRBP^fl/fl^ (*n* = 23), Rho-iCre75 IRBP^fl/fl^ (*n* = 27); Rx-Cre IRBP^fl/fl^ (*n* = 22); and HRGP-Cre Rho-iCre75 IRBP^fl/fl^ (*n* = 11)

The retinas were probed for IRBP mRNA as well as Hypoxanthine phosphoribosyltransferase 1 (Hprt) mRNA as an internal standard (Fig. 1). Gene expression data was presented as a ratio of IRBP/Hprt. Control IRBP^fl/fl^ mice had an IRBP gene expression ratio of 13.2 ± 1.38. HRGP-Cre IRBP^fl/fl^ (cone photoreceptor knockout) mice had a nonsignificant decrease of 10.2% ± 7.6% in gene expression. Chx10-Cre IRBP^fl/fl^ mice affects the retinal progenitor cells (with subsequent impact on bipolar cells) and had a 39% ± 37% drop. The Rho-iCre75 IRBP^fl/fl^ line affects the rod photoreceptors, which make up approximately 97% of photoreceptors in the mouse eye.^40^ These mice experienced an IRBP expression knockdown of 91.1% ± 1.5%. The double knockout of HRGP-Cre Rho-iCre75 IRBP^fl/fl^ had a 98% ± 0.03% loss of IRBP gene expression. The Rx-Cre IRBP^fl/fl^ line affects the entire neural retina. These mice experienced an almost complete knockout (99.7% ± 0.40%) of IRBP expression. This indicates that IRBP expression is proportionally dependent on the amount of each cell type present in the retina. Based on the results from the cells tested (rods, cones, and bipolar cells), the cell type in which IRBP gene expression is made deficient impacts overall IRBP expression (see the Discussion).

**Figure 2.**
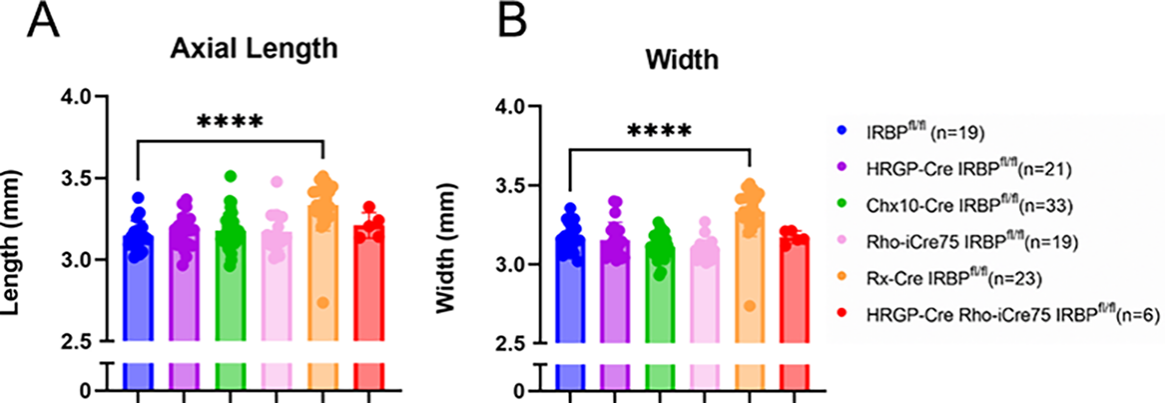
External Eye Measurements show increased eye sizes in Rx-Cre IRBP^fl/fl^ mice. Axial length and width were not significantly different for all lines except Rx-Cre IRBP^fl/fl^ which had significantly longer and wider eyes. One-way ANOVA with Dunnett's multiple comparison's test. * Represents *P* < 0.05; ** represents *P* < 0.01; and *** represents *P* < 0.001. Sample size = IRBP^fl/fl^ (*n* = 27), HRGP-Cre IRBP^fl/fl^ (*n* = 21), Chx10-Cre IRBP^fl/fl^ (*n* = 33), Rho-iCre75 IRBP^fl/fl^ (*n* = 19); Rx-Cre IRBP^fl/fl^ (*n* = 23); and HRGP-Cre Rho-iCre75 IRBP^fl/fl^ (*n* = 5).

To determine whether these mice had a myopic phenotype similar to what we observed in the conventional (traditional) IRBP knockout mouse, postmortem eye dimensions were analyzed (Fig. 2).^23^ External eye dimensions would be irregular for misshapen or elongated eyes. The HRGP-Cre IRBP^fl/fl^ and Chx10-Cre IRBP^fl/fl^ mice had no differences in any eye dimension measurements. The Rho-iCre75 IRBP^fl/fl^ mice had no significant differences in eye length or roundness. The Rx-Cre IRBP^fl/fl^ mice had significant differences in the length and width of the eye. Rx-Cre IRBP^fl/fl^ mice had a 0.18 ± 0.18 mm increase in length and an 0.21 ± 0.15 mm increase in width. HRGP-Cre Rho-iCre75 IRBP^fl/fl^ mice had a no significant increase in length or width. Based on the eye dimensions, the Rx-Cre IRBP^fl/fl^ line is the only one with elongated eye growth and a myopic phenotype.

**Figure 3.**
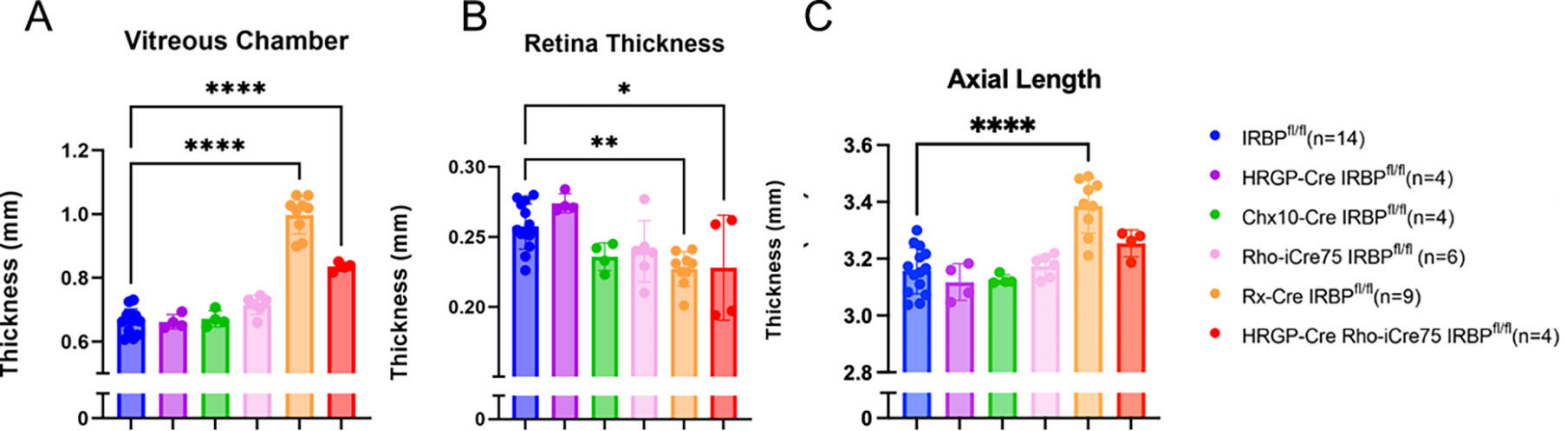
Bioptigen SD4310 SD-OCT whole eye measurements show increased vitreous chamber depth in Rho-iCre IRBP^fl/fl^, HRGP-Cre Rho-iCre75 IRBP^fl/fl^, and Rx-Cre IRBP^fl/fl^ lines similar to what was previously observed in germline IRBP knockout.^22^^,^^23^ One-way ANOVA. * Represents *P* < 0.05; ** represents *P* < 0.01; and *** represents *P* < 0.001. Sample size = IRBP^fl/fl^ (*n* = 14), HRGP-Cre IRBP^fl/fl^ (*n* = 4), Chx10-Cre IRBP^fl/fl^ (*n* = 4), Rho-iCre75 IRBP^fl/fl^ (*n* = 6); Rx-Cre IRBP^fl/fl^ (*n* = 9); and HRGP-Cre Rho-iCre75 IRBP^fl/fl^ (*n* = 4).

To continue to test whether these mice had a myopic phenotype, whole eye biometry was analyzed for each of the five lines (Fig. 3). Previously, it has been shown with whole eye biometry that a germline IRBP^ko/ko^ mouse has significant phenotypic changes, particularly seen in vitreous chamber depth (VCD).^23^ In order to investigate whether this abnormal phenotype was caused by lack of IRBP expression in a particular cell type, six different measurements from the whole eye image were collected: CCT), ACD, LT, VCD, RT, and AL. In the HRGP-Cre, Chx10-Cre, and Rho-iCre75 mouse lines, there were no significant differences seen in any of the 6 measurements. The HRGP-Cre Rho-iCre75 line had a large increase (25% ± 2%) in VCD, but a thinning of RT (11% ± 14% loss). Rx-Cre IRBP lines had a 50% ± 9% increase in VCD as well as in AL (7% ± 3%) with a thinning of RT (12% ± 5%).

**Figure 4.**
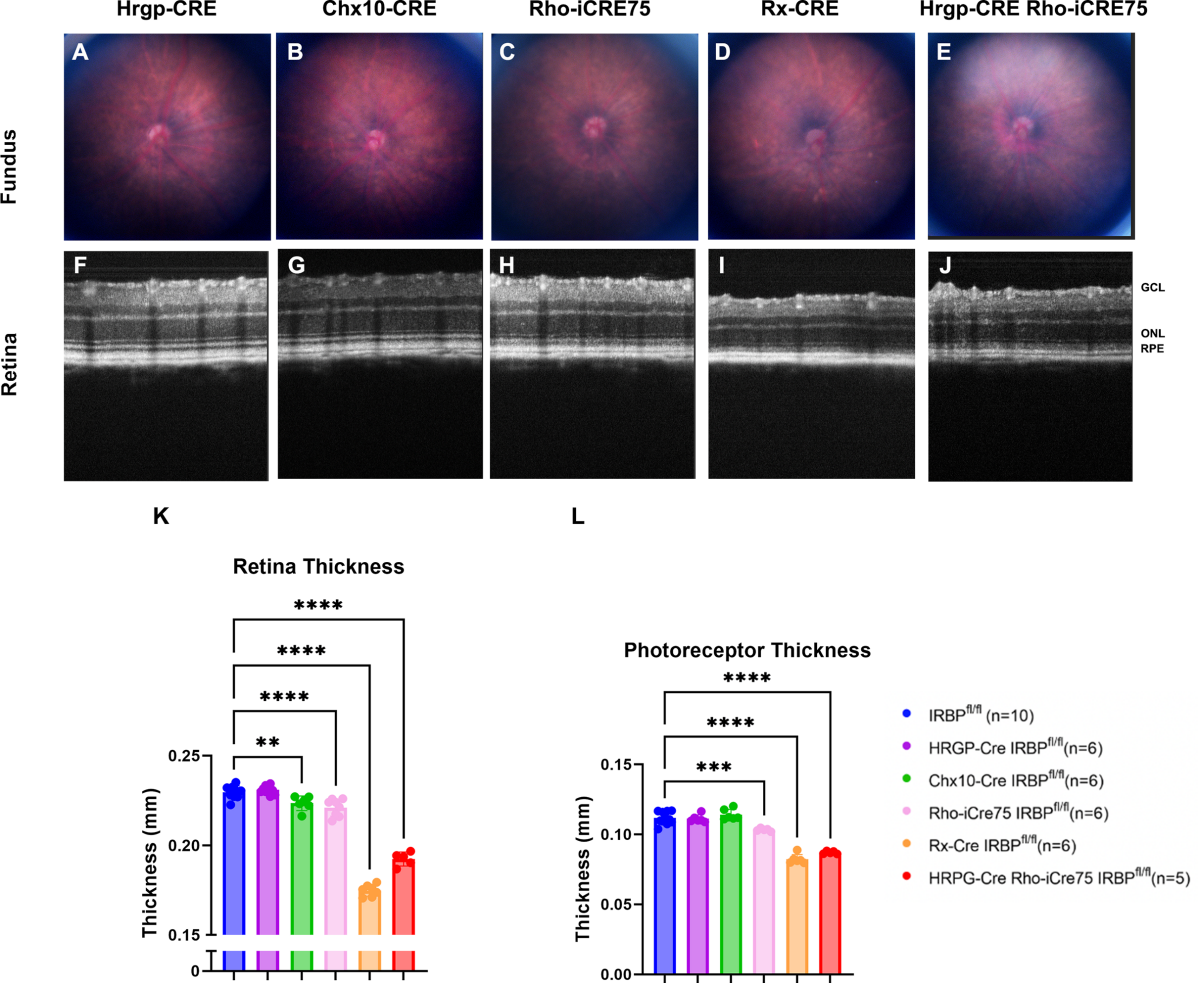
In vivo SD-OCT imaging shows loss of retinal thickness in Rho-iCre IRBP^fl/fl^, HRGP-Cre Rho-iCre75 IRBP^fl/fl^, and Rx-Cre IRBP^fl/fl^ lines. Fundus images show healthy normal fundus in all lines except Rx-Cre IRBP^fl/fl^. Rx-Cre and HRGP-Cre Rho-iCre75 IRBP^fl/fl^ has loss of retina thickness located in photoreceptor layers. These findings indicate a retinal degeneration in Rho-iCre IRBP^fl/fl^, HRGP-Cre Rho-iCre75 IRBP^fl/fl^, and Rx-Cre IRBP^fl/fl^ lines. One-way ANOVA. * Represents *P* < 0.05; ** represents *P* < 0.01; and *** represents *P* < 0.001. Sample size = IRBP^fl/fl^ (*n* = 10), HRGP-Cre IRBP^fl/fl^ (*n* = 6), Chx10-Cre IRBP^fl/fl^ (*n* = 6), Rho-iCre75 IRBP^fl/fl^ (*n* = 6); Rx-Cre IRBP^fl/fl^ (*n* = 6); and HRGP-Cre Rho-iCre75 IRBP^fl/fl^ (*n* = 5).

In vivo SD-OCT imaging was used to look for retinal deficits. Retinal degeneration is a key phenotype in the germline IRBP^ko/ko^ mouse.^22^^,^^23^^,^^25^ Shown in Figure 4, the HRGP-Cre had no significant differences in retina thickness, photoreceptor thickness, or fundus imaging. These results suggest normal retina health. No damage was present in the fundus image of the Rho-iCre75 IRBP^fl/f^ mouse. However, the retina thickness was reduced significantly, by approximately 9 ± 5.9 microns. The fundus of the HRGP-Cre Rho-iCre75 IRBP^fl/f^ mouse looked normal and healthy, but the retina had a significant loss of thickness by 37 ± 5.3 microns. The Rx-Cre IRBP^fl/f^ mouse also showed a significant decrease in retina thickness. The entire retina was significantly thinner (by loss of approximately 54 ± 4.8 microns), and the photoreceptor layer was also substantially thinner. The inner segments, outer segments, and retinal pigmented epithelium of this line had auto fluorescent reflection. The fundus images had white spots across the retinas, indicating damage.

**Figure 5.**
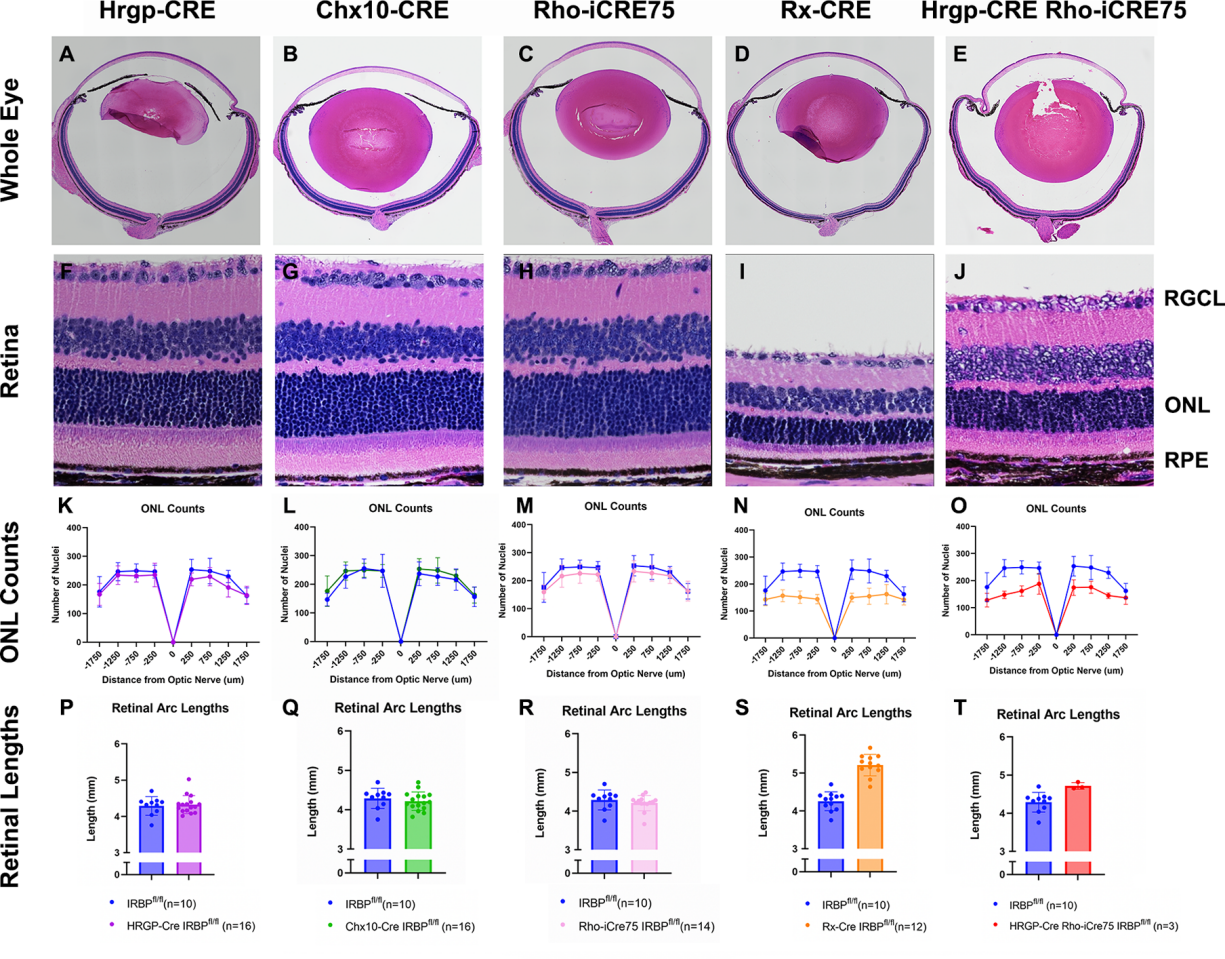
H&E images showing retinal morphology as well as quantifications of nuclei and retinal arc lengths. HRGP-Cre IRBP^fl/fl^, Chx10-Cre IRBP^fl/fl^, and Rho-iCre75 IRBP^fl/fl^ retinas show normal morphology with no obvious differences. When quantified, Rho-iCre75 IRBP^fl/fl^ mice showed a slight decrease in ONL counts. The Rx-Cre IRBP^fl/fl^ line show a clear loss of nuclei and inner and outer segments in morphology. The nuclear count results confirm results in Figure 4 showing loss of photoreceptor cells. The retinal arc length measurements confirm axial length as previously shown Figure 2. Retinal arc lengths were *t*-tests. ONL counts were Tukey's mixed-effects comparisons. * Represents *P* < 0.05; ** represents *P* < 0.01; and *** represents *P* < 0.001. Sample size = IRBP^fl/fl^ (*n* = 13), HRGP-Cre IRBP^fl/fl^ (*n* = 11), Chx10-Cre IRBP^fl/fl^ (*n* = 13), Rho-iCre75 IRBP^fl/fl^ (*n* = 14); Rx-Cre IRBP^fl/fl^ (*n* = 19); and HRGP-Cre Rho-iCre75 IRBP^fl/fl^ (*n* = 3).

H&E staining was conducted on sagittal sections of eyes from each of the mouse lines (Fig. 5). There were no obvious morphological differences in either the HRGP-Cre or Chx10-Cre lines (see Figs. 5F, 5G). These lines also showed no differences in ONL nuclei counts (see Figs. 5K, 5L), suggesting that IRBP gene expression in cones and bipolar cells has little effect on retinal health. In the Rho-iCre75 IRBP^fl/f^ mice, the retinal arc lengths were normal (see Fig. 5R), further confirming a lack of myopia. They also had no statistically significant morphometric differences, although the ONL counts trended lower than IRBP^fl/fl^ (see Fig. 5M). The Rx-Cre IRBP^fl/f^ mice had significant differences in morphology (see Fig. 5I). There were half as many nuclei in the ONL compared to IRBP^fl/f^ (see Fig. 5N). The retinal arc lengths were substantially longer (see Fig. 5S), corroborating the increased eye size seen with the Bioptigen SD4310 SD-OCT imaging and external eye dimensions.

**Figure 6.**
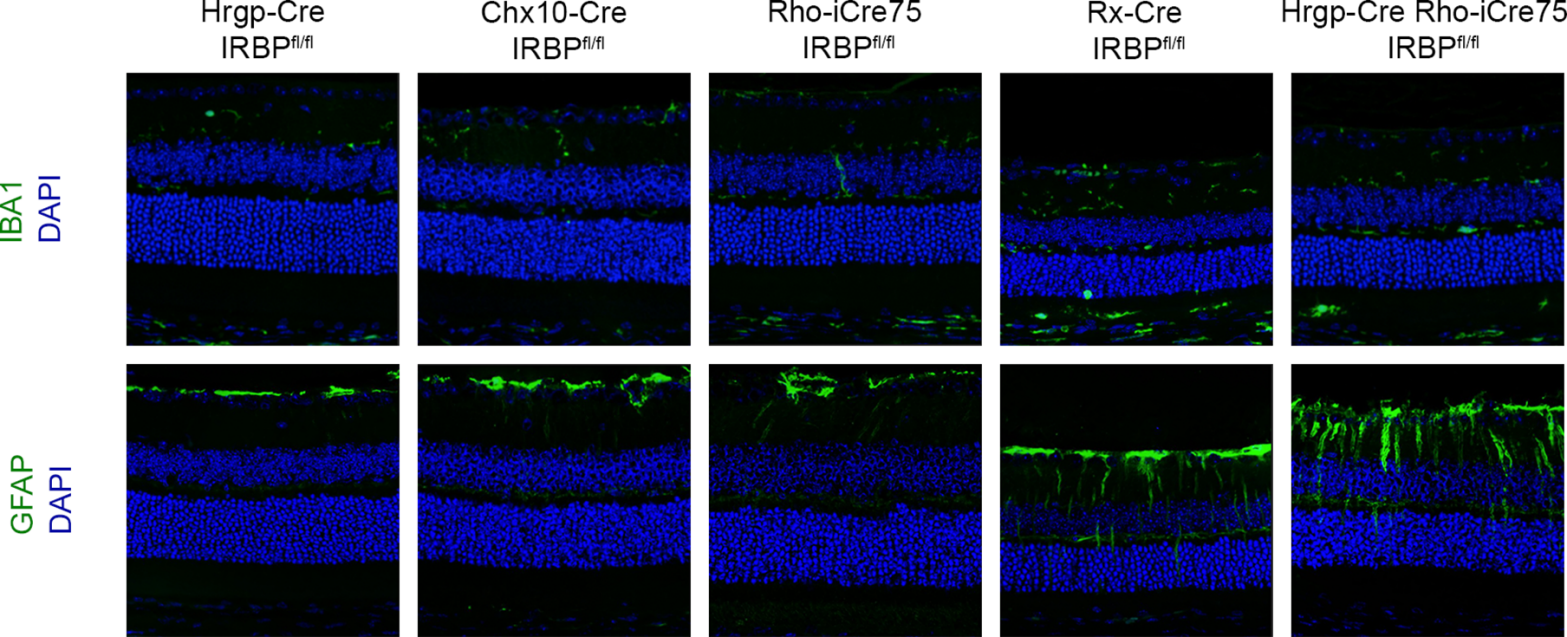
Immunofluorescence staining shows IBA1 and GFAP staining throughout all layers in retina on Rx-Cre IRBP^fl/fl^ and Hrpg-Cre Rho-iCre75 IRBP^fl/fl^. HRGP-Cre IRBP^fl/fl^, Chx10-Cre IRBP^fl/fl^, and Rho-iCre75 IRBP^fl/fl^ have normal distribution of IBA1 and GFAP staining. Rx-Cre IRBP^fl/fl^ and HRGP-Cre Rho-iCre75 IRBP^fl/fl^ have extended GFAP and IBA1 expression in the outer nuclear layer, indicating cellular and immune response to photoreceptor cell death. Sample size = IRBP^fl/fl^ (*n* = 14), HRGP-Cre IRBP^fl/fl^ (*n* = 8), Chx10-Cre IRBP^fl/fl^ (*n* = 8), Rho-iCre75 IRBP^fl/fl^ (*n* = 8); Rx-Cre IRBP^fl/fl^ (*n* = 8); and HRGP-Cre Rho-iCre75 IRBP^fl/fl^ (*n* = 8).

Retina sections were also stained for IBA-1 expression as a marker for microglia and macrophage differences in the retina (Fig. 6). As seen previously, HRGP-Cre IRBP^fl/fl^, Chx10-Cre IRBP^fl/fl^, and Rho-iCRE75 IRBP^fl/fl^ lines showed normal morphology of macrophages and microglia (see Figs. 6A, 6B, 6C). The Rx-Cre IRBP^fl/fl^ line had significant IBA-1 staining throughout the entire retina with a large quantity in the subretinal space (see Fig. 6D). The double knockout of HRGP-Cre+Rho-iCre75 IRBP^fl/fl^ had an increase of macrophages and microglia that migrated to the subretinal space, suggesting an inflammatory response following possible damage to photoreceptors and RPE (see Fig. 6E). GFAP staining was done to look at retinal stress in the lines. HRGP-Cre IRBP^fl/fl^, Chx10-Cre IRBP^fl/fl^, and Rho-iCRE75 IRBP^fl/fl^ lines all showed normal GFAP staining in the ganglion cell layer (GCL; see Figs. 6F, 6G, 6H). Rx-Cre IRBP^fl/fl^ had GFAP expression throughout the IPL and OPL (see Fig. 6I). HRGP-Cre Rho-iCre75 IRBP^fl/fl^ displayed increased GFAP expression in the IPL and OPL (see Fig. 6J).

**Figure 7.**
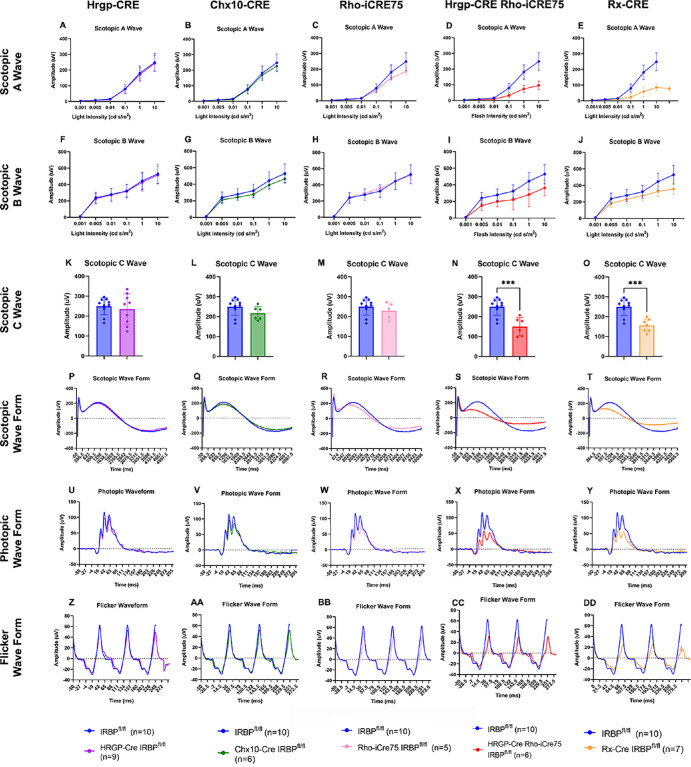
Electroretinograms showing significant loss of function in Rx-Cre and HRGP-Cre Rho-iCre75 lines. The HRGP-Cre IRBP^fl/fl^, Chx10-Cre IRBP^fl/fl^, and Rho-iCre75 IRBP^fl/fl^ lines showed no to small decreases in scotopic, photopic, and flicker ERGs. Rx-Cre IRBP^fl/fl^ and HRGP-Cre Rho-iCre75 IRBP^fl/fl^ lines had significant decreases of roughly 50% to 60% in scotopic A and C waves and photopic and flicker waveforms. These findings confirm photoreceptor cell death and loss of function. Two-way ANOVA, Sidak's multiple comparison test. Sample size = IRBP^fl/fl^ (*n* = 10), HRGP-Cre IRBP^fl/fl^ (*n* = 9), Chx10-Cre IRBP^fl/fl^ (*n* = 3), Rho-iCre75 IRBP^fl/fl^ (*n* = 8); Rx-Cre IRBP^fl/fl^ (*n* = 7); and HRGP-Cre Rho-iCre75 IRBP^fl/fl^ (*n* = 6).

Retinal function was assessed using full-field ERGs (Fig. 7). Mice were tested in scotopic and photopic light conditions. In both the HRGP-Cre IRBP^fl/fl^ and Chx10-Cre IRBP^fl/fl^ mice, the scotopic, photopic, and flicker ERGs all showed little to no difference at any flash intensity compared to IRBP^fl/fl^. These results suggest that these mice have no detectable retinal functional deficits. In the Rho-iCre75 IRBP^fl/fl^ line, there was a reduction in the a- and c-wave at the highest flash intensity in scotopic conditions (see Figs. 7C, 7M) suggesting loss of rod photoreceptor function. However, there was no change in the b-wave, indicating normally functioning bipolar cells (see Fig. 7H). There was also a decrease by approximately 20% in the photopic and flicker protocols (see Figs. 7W, 7BB). The Rx-Cre IRBP^fl/fl^ line had a substantial loss of function, reflected in all ERGs. Across all tests, this line experienced a 50% decrease at the highest flash intensities.

## Discussion

The results of this study provide insights into the role of IRBP in retinal development,^18^^,^^19^^,^^41^^–^^43^ health, and function, revealing the importance of the location, quantity, and timing of IBRP expression. We determined this by using various cell-type-specific Cre drivers that express Cre only at known time points and in cell-types (specified by the given promoter that was driving Cre expression) to knock out IRBP selectively. Our end point, postnatal day 30, allowed for investigation of a stage of maturation, because we were looking for robust myopia, functional deficits, and retinal morphology changes.

The most significant findings here is that IRBP expression in both rods and cones is needed for full retinal function and correct morphological development that prevents retinal degeneration or myopia. Thus, a bit surprisingly, it appears that cone expression of IRBP can play a role in partially maintaining retina morphology and function and in maintaining ocular morphometrics that permit emmetropic stability.

IRBP is expressed in various cell types in the eye. Large quantities of IRBP mRNA were found in the photoreceptors, but there is also expression found in three subtypes of bipolar cells.^20^^,^^21^^,^^44^^,^^45^ The role of IRBP in these bipolar cells is unknown. To determine the possible effect of IRBP in this group, a bipolar-specific knockout was used, Chx10-Cre. However, after knockout, there was no change in any significant measure compared to a wildtype mouse at postnatal day 30. This could be due to relatively little IRBP that is expressed in the bipolar cells that could cause any significant changes. It is possible that the vast amount of IRBP from the rods and cones are sufficient to compensate for loss in bipolar cells. A similar effect was observed for the cone-specific knockout line, HRGP-Cre, suggesting the IRBP produced from cones may not be substantial enough to affect retina and eye development.

Our hypothesis for the rod-specific knockout, Rho-iCre75 IRBP^fl/fl^, anticipated no change in eye size. Rod photoreceptors do not appear until later in eye growth, originating from late progenitor cells.^46^ In addition, IRBP mRNA has a great increase of expression from postnatal day 1 to postnatal day 9^18^ in whole retina. The Rho-iCre75 driver has been shown to express functionally adequate levels of Cre at postnatal day 11, but do not reach maximum levels until 2 months.^31^ From previous IRBP knockout studies, myopia begins at postnatal day 7 and the myopic shift plateaus at postnatal day 12.^22^ Because myopia starts earlier in development than the Rho-iCre75 driver expresses Cre, the late knockout through rod photoreceptors should not impact eye size, if myopia and retinal degeneration are independent. This could be attributed to IRBP-dependent normal eye growth regulation already being substantially completed before a lack of rod-specific IRBP expression could begin. Retinal degeneration in the IRBP^ko/ko^ mouse model occurs much later in the eye development, starting at postnatal day 25.^22^ The Rho-iCre75 IRBP^fl/fl^ mice displayed no change in ONL nuclei. The ERGs showed a 25% drop in function at the postnatal day 30 time point, a trend, but not statistically significant (*P* = 0.0557), which is possibly too early to catch significant changes. Rho-iCre75 IRBP^fl/fl^ retinas were thicker compared to the Rx-Cre IRBP^fl/fl^ and HRGP-Cre+Rho-iCre75 IRBP^fl/fl^. The better retina morphology and function could be explained by the absence of myopia development or that the remaining IRBP produced from the cone photoreceptors is enough to compensate for retina loss.

The use of the cone and rod double knockout, HRGP-Cre+Rho-iCre75 IRBP^fl/fl^, was aimed to investigate whether the remaining rod or cone IRBP in the single knockout lines could be enough to offer some protection. When IRBP is knocked out in both cell types, there would be IRBP produced in the bipolar cells alone. There was significant myopia, loss of retinal thickness, and function that suggests that IRBP plays a vital role during development for eye size determination. The study highlights the necessity of IRBP in some capacity from photoreceptors for the retina to properly develop. The IRBP levels seen in the double cone and rod knockout were equivalent to those seen in a complete IRBP knockout, indicating that the proportion of IRBP loss in photoreceptors is directly proportional to loss of retinal thickness. Similarly, a complete retinal knockout, Rx-Cre IRBP^fl/fl^, had almost identical responses as the HRGP-Cre+Rho-iCre75 IRBP^fl/fl^ and the germline IRBP^ko/ko^ mice, reinforcing the critical role of IRBP in retinal health.

**Figure 8.**
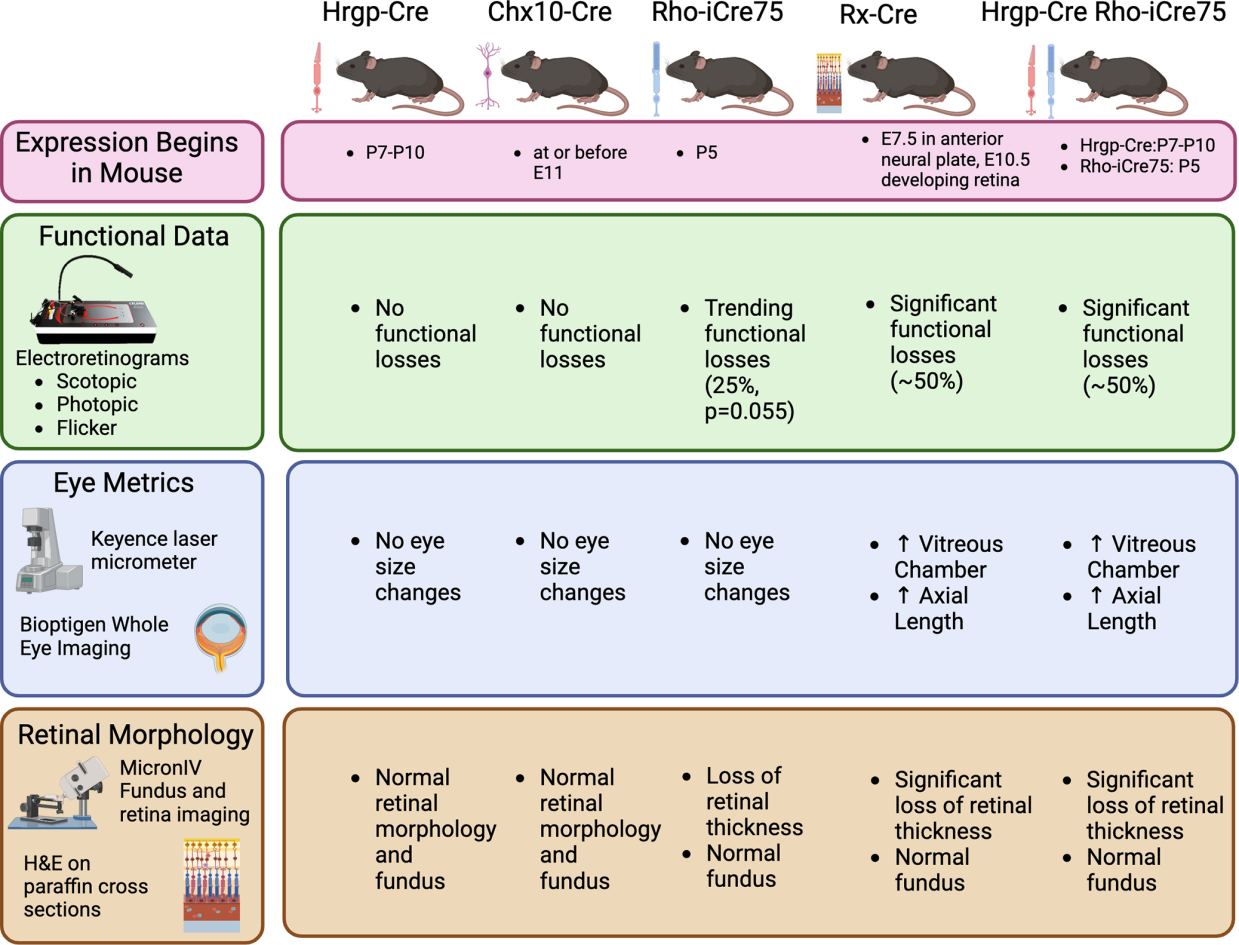
Summary figure presenting major results for all mouse lines. Chx10 driver expression begins at or before E11.5. Rx expression starts at E7.5 in anterior neural plate, and E10.5 in developing retina. Rho expression begins at postnatal day 5, and Hrgp starts between postnatal day 7 and postnatal day 10. All experiments were conducted with mice between postnatal day 28 and postnatal day 32. Created with BioRender.com.

In conclusion, near-complete loss of IRBP results in myopia, disorganized morphology, and loss of function (Fig. 8). Although bipolar cells do express IRBP, loss of IRBP in these cell types did not have a noticeable effect, warranting further exploration of the role of IRBP in bipolar cells. Moreover, timing of IRBP loss played an important role in eye development, with the Rho-iCre75 IRBP^fl/fl^ late induction of IRBP gene deletion causing a small retinal degeneration and loss of some a-wave (25%) without the previously observed increase in AL.^31^ This implies two separate and independent mechanisms of action for IRBP function. Future investigations to overexpress or misexpress IRBP might aid in determining mechanisms by which IRBP acts.

### Limitations

Hprt was the gene we used as the reference gene for ddPCR^47^^–^^49^; Hprt could potentially be a confound if it were to vary systemically. This research was limited by having a single end point at postnatal day 30; future experiments could investigate outcomes at other ages to assess whether retinal degenerations progress as in human retinitis pigmentosa, specifically RP66.

### Future Work

We speculate that the different roles of IRBP on myopia and retinal degeneration may involve different ligands (perhaps including retinoic acid) bound to IRBP early in development that drive myopia and eye size, whereas the later effects of IRBP in preventing retinal degeneration may involve a different set of the ligands (including all-trans-retinol and 11-cis-retinal) integral to the vitamin A cycle. We hope to test these ideas in future work. This research leads to a deeper understanding of IRBP's multifaceted role in retinal development and its potential as a target for therapeutic interventions.

## Supplementary Material

Supplement 1

Supplement 2
