# Supplementary material for: Conditional Knockouts of Interphotoreceptor Retinoid Binding Protein Suggest Two Independent Mechanisms for Retinal Degeneration and Myopia
Source: Invest Ophthalmol Vis Sci. 2024 Jun 21;65(6):32. doi: 10.1167/iovs.65.6.32 (PMC11193143; doi:10.1167/iovs.65.6.32)
Supplement: Supplement 1 [file iovs-65-6-32_s001.docx]

Supplement contents.

Supplemental figures S1 and S2.

Word document that includes the captions for S1 and S2. [This document].

A text file that contains the sequence of the IRBP gene with the two loxP sites and modifications.


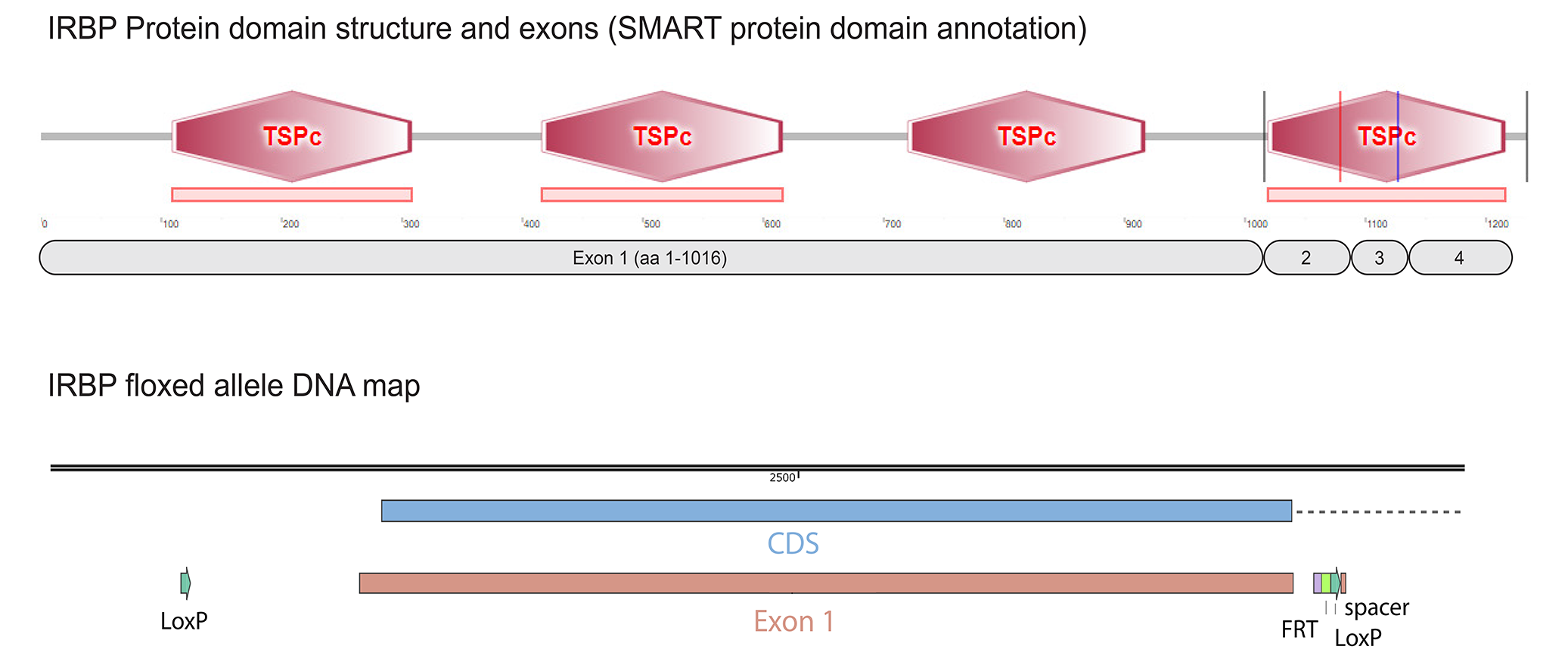


Figure S1. The amino acid motif analysis of the IRBP protein and the corresponding locations of splice sites from the RBP3 gene.


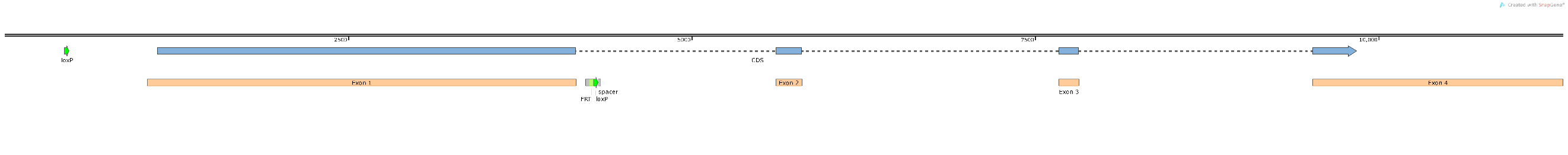


Figure S2. The DNA sequence of the IRBP (RBP3) gene with the loxP sites introduced into the sequence. The positions of the Transcription Start Site (TSS) are indicated on the second line.

The positions of exons and introns are noted. The upstream loxP is located about 600 nucleotides 5’ to the TSS, which indicates that the IRBP promoter is deleted once Cre acts to delete sequences between the two loxP sites.
